# Effects of glucose concentration on 1,18-*cis*-octadec-9-enedioic acid biotransformation efficiency and lipid body formation in *Candida tropicalis*

**DOI:** 10.1038/s41598-017-14173-7

**Published:** 2017-10-23

**Authors:** Irina Funk, Volker Sieber, Jochen Schmid

**Affiliations:** 1 Technical University of Munich, Chair of Chemistry of Biogenic Resources, Schulgasse 16, 94315 Straubing, Germany; 2 Catalysis Research Center, Technical University of Munich, 85748 Garching, Germany

## Abstract

The unsaturated long-chain α,ω-dicarboxylic acid 1,18-*cis*-octadec-9-enedioic acid (*cis*-ODA) is a versatile precursor of various valuable compounds, such as polymers, and can be obtained from renewable resources. This makes *cis*-ODA highly attractive for the chemical industry where there is a growing interest in sustainable processes. However, chemical synthesis of the *cis* isomers is currently not feasible. In contrast, biotechnological production allows for highly specific and selective reactions. Therefore, we developed an efficient production strategy for *cis*-ODA using *Candida tropicalis* as a whole-cell biocatalyst for the biotransformation of oleic acid, which naturally occurs in various fats and oils. Applying a bench-top system comprising eight parallel bioreactors, the production process was characterised and optimised for high productivity. Glucose feed rate was identified as the most crucial process parameter influencing product yield, with high rates inducing oleic acid incorporation into triacylglycerols and storage in lipid bodies. Conversely, application of medium-chain length fatty acid as a substrate did not show any occurrence of lipid bodies. Applying the lowest possible molar ratio of glucose to oleic acid (1.5) resulted in marginal lipid body formation, but led to a peak volumetric productivity of 0.56 g/L/h and a final titre of approximately 45 g/L with a corresponding yield of 70%.

## Introduction

Long-chain α,ω-dicarboxylic acids (DCAs) are important precursors in the chemical industry since they can be used for producing numerous products, such as polyamides, fragrances and lubricants^1,2^. The chemical synthesis of long-chain DCAs is challenging because of the complex production process and the high potential of producing undesired by-products. The production of unsaturated long-chain DCAs is demanding, especially due to the chemical reactivity of the double bond, which can lead to numerous by-products as well as migration of the double bond and configurational changes^1^. Unsaturated long-chain DCAs serve as highly useful precursors for producing bio-based cross-linked polymers and can subsequently be modified further, like the epoxidation the double bond^2,3^. In this regard, 1,18-octadec-9-enedioic acid (ODA) is a particularly interesting molecule that can be produced by targeted functionalisation of renewable raw materials. Although ODA can be chemically synthesised by self-metathesis of oleic acid catalysed by second-generation Grubbs catalysts, only the *trans* isomer production has been reported so far (molar yield of approximately 50%)^3–5^. Because enzymatic conversion is more selective and can be achieved under reaction conditions milder than current chemical methods, this represents an alternative production route and leads to preservation of the *cis* configuration. *Candida tropicalis* ATCC 20962 is frequently used for the biotechnological production of long-chain α,ω-dicarboxylic acids from *n*-alkanes or fatty acids (FAs) via ω-oxidation. This yeast strain is genetically modified by disrupting the *pox4/pox5* genes (coding the acetyl-CoA oxidase) to eliminate the ability of the yeast to utilise FAs as a carbon source^1^. Applying *C. tropicalis* as a whole-cell biocatalyst for producing 1,18-*cis*-octadec-9-enedioic acid (*cis*-ODA) from oleic acid has been described by several groups^1,6–9^. However, few such studies have systematically examined production performance under varying process parameters.

Due to the utilisation of highly hydrophobic substrates, inhomogeneity represents the main limitation for bio-based long-chain DCA production. Although this inhomogeneity problem can be solved by an alkaline pH shift to enhance substrate and product solubility^10^, the resulting alkaline stress may cause metabolic stress and ultimately reduce cell viability. Therefore, designing an appropriate pH shift is essential but is usually not considered in *cis*-ODA production studies. Further, the media components, especially glucose, have a significant influence on product formation in certain biotransformation approaches^8^. Glucose was described to have a negative effect on the ω-oxidation pathway due to catabolite repression^11–13^ and inhibitory effects on the induction of the alkene and DCA-producing enzymes^2,14^. To the best of our knowledge, there are no detailed investigations on utilisation of oleic acid by *C. tropicalis* ATCC 20962 and dependence on glucose levels for *cis*-ODA production. Therefore, the negative effects of elevated glucose levels and the maximum applicable glucose feed rate for a given amount of biomass are still open questions. To clarify the influence of glucose for optimal *cis*-ODA production, we conducted bioconversion studies using *C. tropicalis* ATCC 20962 in a parallel bioreactor bench-top system (8 × 1 L) in combination with transcriptional profiling of the genes involved in bioconversion.

## Results and Discussion

The production process of *cis*-ODA relies on whole-cell biotransformation of oleic acid by *C. tropicalis* and therefore can be separated into growth and biotransformation phases. The growth phase must yield sufficient biocatalysts in form of cell biomass for subsequent conversion of oleic acid in the biotransformation phase. To enable efficient and reliable production of *cis*-ODA, both phases were optimised using a bench-top bioreactor system consisting of eight parallel bioreactors.

### Optimisation of the growth phase

The initial step for an efficient biotransformation process is the production of high amounts of biomass in the growth phase^8^. In general, the total amount of biomass represents the theoretical number of active biocatalysts for whole-cell biotransformation. To achieve enhanced biomass production in the growth phase a fed-batch operational mode was applied. This led to almost twofold higher final cell biomass of 22.5 ± 2.3 g/L at the end of the growth phase when compared to the batch process (Table 1). Conversely, applying a fed-batch approach by additional glucose supply resulted in a biomass yield coefficient (Table 1) slightly lower than the theoretical maximal value of 0.5 g/g based on the aerobic metabolism of glucose^15^, indicating possible occurrence of the Crabtree effect. Therefore, exemplarily samples obtained throughout the whole process were analysed for ethanol production. This analysis revealed an average ethanol concentration of 3.2 ± 0.6 g/L (n = 4) at the end of the growth phase confirming the channelling of glucose in a fermentative pathway. To reduce ethanol accumulation and enhance biomass production further a continuous glucose supply at low feed rate will be a suitable approach and is the focus of upcoming studies.

**Table 1:** Overview of growth phase parameters in batch mode versus fed-batch mode with and without pH shift.

Operational mode		Cell biomass [g/L]	Colony forming units [CFU/mL]	Specific growth rate [h^−1^]	Doubling time [h]	Biomass yield coefficient [g_biomass_/g_glucose_]
Batch*		14.3 ± 0.2	—	0.14 ± 0.02	5.18 ± 0.62	0.44 ± 0.01
Fed-Batch	Without pH shift**	22.5 ± 2.3	6.01 × 10^8^ ± 1.47 × 10^8^	0.26 ± 0.02	2.69 ± 0.22	0.37 ± 0.03
	With pH shift**	24.8 ± 2.2	8.53 × 10^8^ ± 1.85 × 10^8^	0.26 ± 0.05	2.72 ± 0.50	0.39 ± 0.04
Ǿ Fed-Batch***		23.6 ± 2.5	6.95 × 10^8^ ± 2.03 × 10^8^	0.26 ± 0.04	2.71 ± 0.38	0.38 ± 0.04

The data represent the mean ( ± standard deviation) at the end of the growth phase (16 h) of independent experiments performed using DASGIP 8 × 1 L parallel bioreactor system as described in methods. Glucose supply for a final concentration of 30 g/L: batch at 0 h, fed-batch at 0 h and 8 h. Sample size: *n = 3, **n = 10, ***n = 20. Ǿ fed-batch represents the mean value of both fed-batch approaches (with and without pH shift).

**Figure 1:**
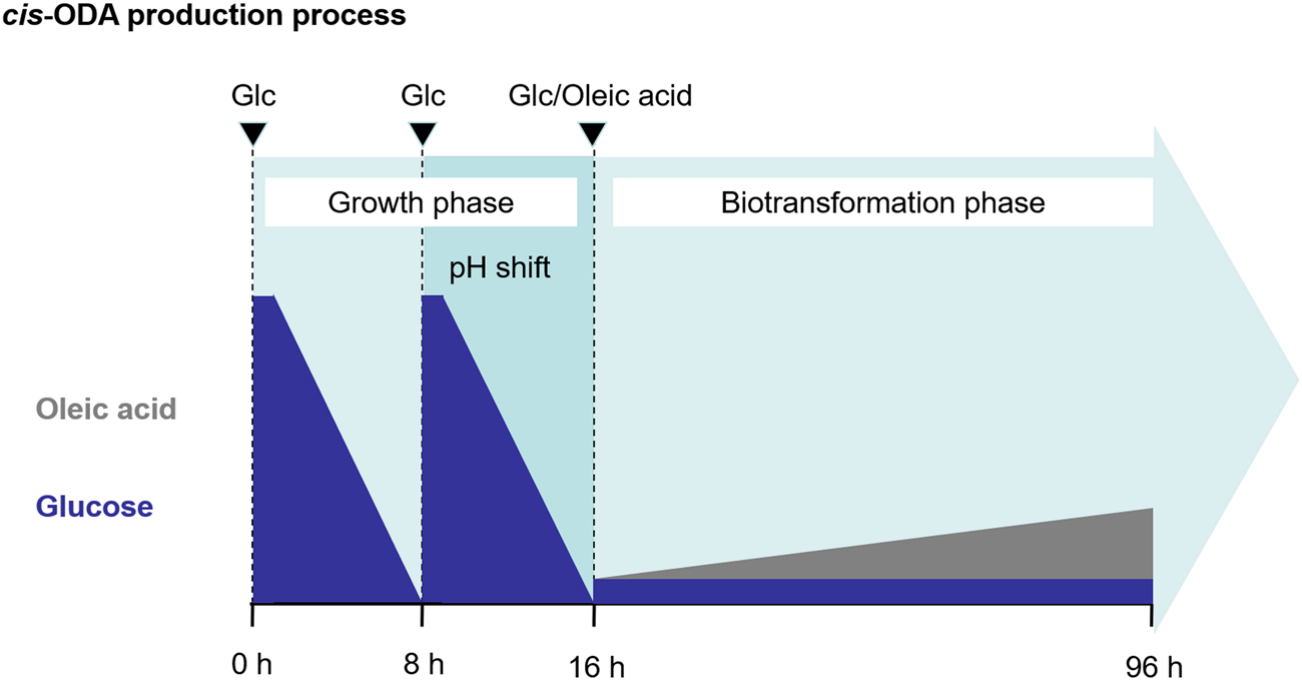
Schematic overview of the optimised *cis*-ODA production process designed in this study. The process is divided into two phases: growth and biotransformation. The growth phase includes glucose supply for a final concentration of 30 g/L at 0 h and 8 h, as well as a pH shift from 5.8 to 8.0 within 6 h starting at 8 h. The biotransformation phase is initiated after 16 h (pH 8.0) by simultaneously starting oleic acid (1 g/L/h) and glucose feeding (0.4–1.25 g/h).

Next to maximization of the biocatalyst amount for the bioconversion process, the growth phase was monitored using several predetermined reaction conditions, in particular, adjustment of pH. Generally, a shift in the pH towards basic conditions is necessary for efficient solubility of fatty and dicarboxylic acids in aqueous medium. This would enhance productivity and is usually applied at the beginning of the biotransformation phase^1,7–10,16,17^. However, as observed in the initial set experiments, simultaneous application of the pH shift and oleic acid feed in the beginning of the biotransformation phase led to a moderate conversion and precipitation of oleic acid onto the walls and installations of the bioreactors at pH of approximately 7.0. A further increase of the pH to 8.0 resulted in initiation of re dissolution of the oleic acid precipitates and enhanced the production of *cis*-ODA (Supplementary Fig. S1). These results indicated a clear dependence of oleic acid solubility on the pH value. Thus, it is essential to initiate oleic acid feed at slightly basic conditions (pH = 8.0). Therefore, to facilitate efficient conversion of oleic acid, the biotransformation phase was initiated at a pH of 8.0 by transferring pH shift into the growth phase (Fig. 1).

**Figure 2:**
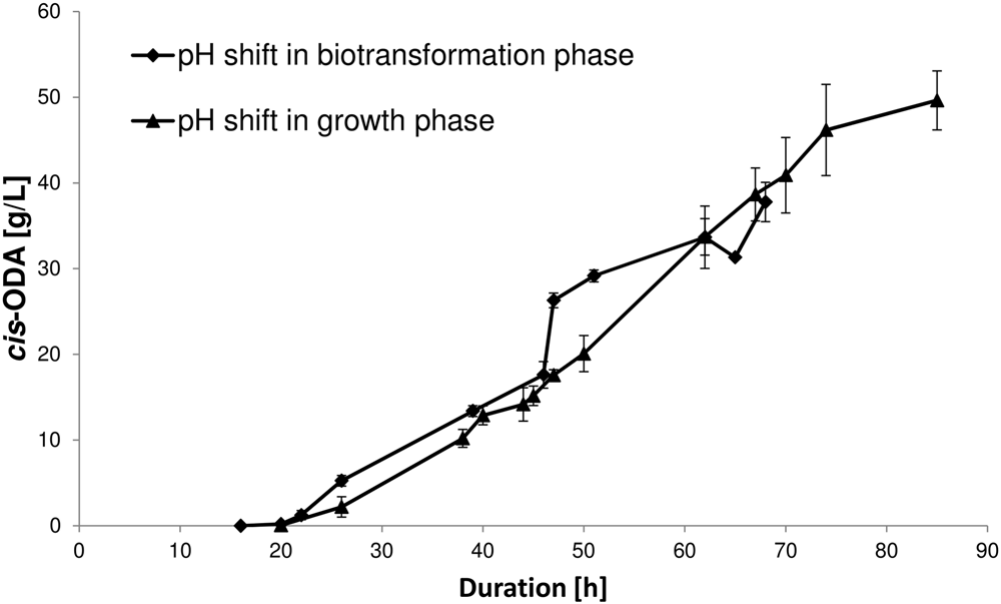
Modulation of 1,18-*cis*-octadec-9-enedioic acid production by changing the timing of the pH shift. These experiments were performed at least in duplicate using the DASGIP 8 × 1 L parallel bioreactor system as described in the methods. The error bars represent the standard deviation.

However, pH shift in the growth phase resulted in no substantial improvement of oleic acid conversion, with product concentration reaching approximately 40 g/L after 68 h, similar to previous experiments when the pH shift was performed during the biotransformation phase (Fig. 2). Nonetheless, increased oleic acid solubility from the beginning of biotransformation phase led to a visible reduction in oleic acid precipitate and therefore allowed for more reliable characterisation of the production process (Fig. 2). Moreover, cell growth and viability were not negatively affected by application of a pH shift in the growth phase, and final cell dry weight of 24.8 ± 2.2 g/L was similar to that achieved during the growth phase without a pH shift (Table 1).

To summarise, modification of the growth phase by applying a fed-batch mode and a controlled pH shift led to enhanced cell biomass production and to slightly basic pH, respectively, providing an optimal starting point for subsequent biotransformation of oleic acid. Analysis of the growth phase showed that the application of a bench-top parallel bioreactor system is suitable for this study and offers reliable and highly reproducible results (Table 1).

### Optimisation of the biotransformation phase

The biotransformation phase is initiated by simultaneous start of glucose feeding as a carbon source to maintain cell metabolism and oleic acid feeding as the substrate for conversion (Fig. 1). Both feed rates represent critical parameters for optimisation of productivity.

### Glucose feed rate: crucial parameter affecting *cis*-ODA productivity

Since this biotransformation approach uses a genetically modified *C. tropicalis* strain (Δ*pox*) incapable of metabolising FAs as a carbon source to produce dicarboxylic acids, an alternative energy source is necessary. As the most common carbon source, glucose is metabolised by many microorganisms and provides sufficient energy to maintain cell metabolism. However, due to the possible occurrence of the Crabtree effect or inhibitory effects of glucose on alkene-degrading and dicarboxylic acid-producing enzymes^2,14^, glucose feed rate is a crucial parameter requiring optimisation. Therefore, various glucose feed rates were applied in continuous operation mode, ranging from 0.4 to 1.25 g/h (refer to methods), and the effects on cell growth and biotransformation were investigated.

**Figure 3:**
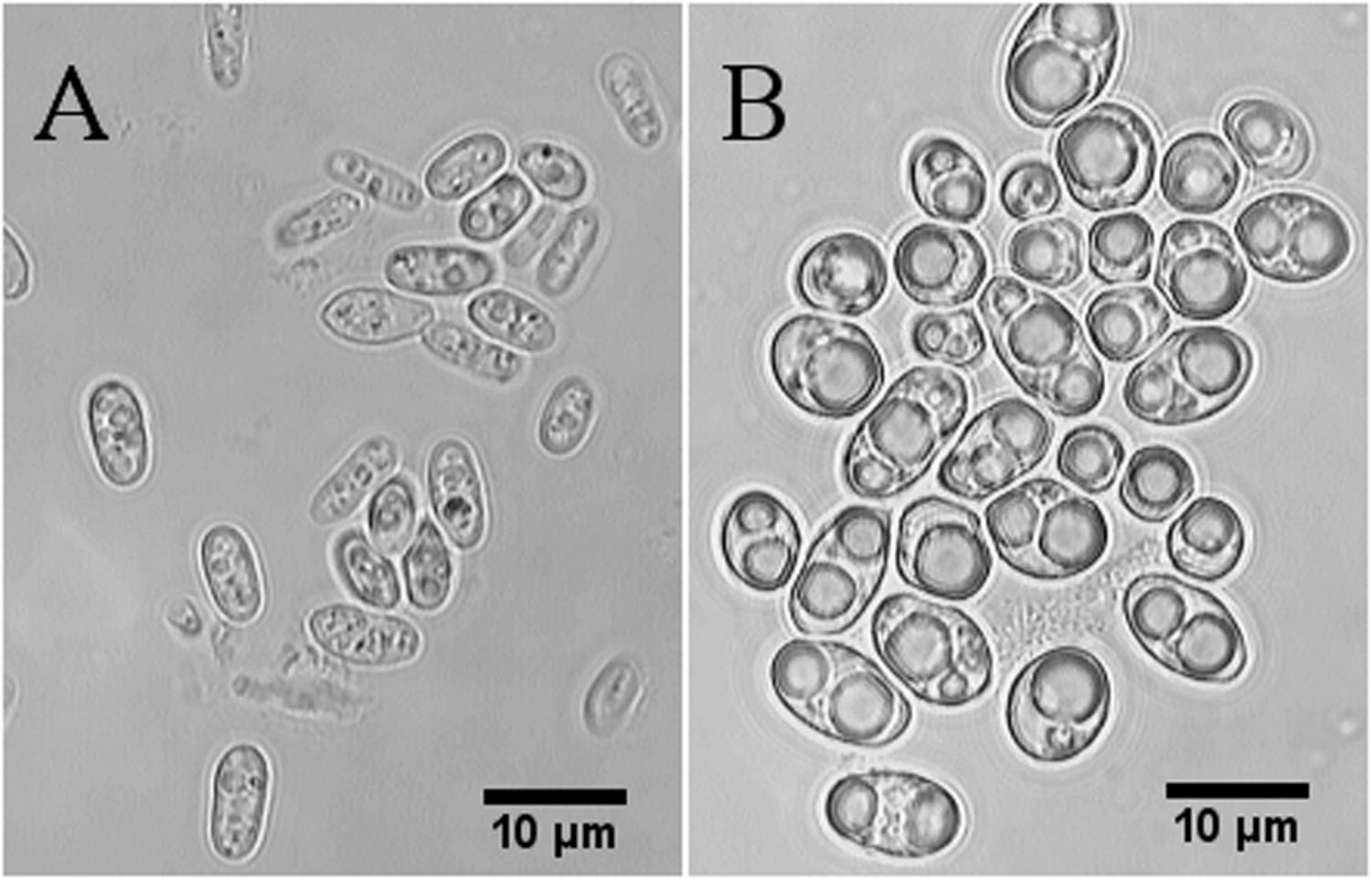
Microscopic views at 1,000 × magnification of *C. tropicalis* after 62 h of the production process. (**A**) Glucose feed at 0.4 g/h, cell length: 6.5 ± 0.9 µm, area: 17.5 ± 4.9 µm^2^ (n = 22). (**B**) Glucose feed at 1.25 g/h, cell length: 7.3 ± 2.0 µm, area: 33.6 ± 12.3 µm^2^ (n = 31). The data represent the mean (±the standard deviation) obtained from cell measurements using ImageJ software. Sample size n is giving in brackets. Figure was edited as described in methods, for original figure refer to Supplementary Information.

As expected, higher glucose feed rate resulted in enhanced biomass production, reaching a final cell dry weight of 118.3 ± 1.3 g/L at feed rate of 1.25 g/h or approximately four times higher than at the lowest glucose feed rate of 0.4 g/h (Table 2). However, enhancing the number of biocatalysts did not lead to higher productivity; rather, increased glucose feed rate drastically decreased product yield, while the conversion of oleic acid remained at almost 100% throughout the whole process (Supplementary Fig. S2, Table 2). This observation indicates the existence of an alternative oleic acid metabolic pathway aside from ω-oxidation. Microscopic investigation to monitor *Candida* cell morphology during production provided clues to the underlying mechanism. Yeast cells exposed to a high glucose feed rate (1.25 g/h) were on average double the size of cells exposed to a lower glucose feed rate and contained vesicles 3.7 ± 0.9 µm (n = 43) in diameter (Fig. 3). The formation and chemical nature of these vesicles were investigated to elucidate possible mechanisms underlying the influence of glucose on product yield.

**Table 2:** Parameters calculated at various glucose feed rates after 68 h of production (52 h of biotransformation).

**Glucose feed [g/h]**	**Final cell biomass [g/L]**	**Colony forming units [CFU/mL]**	**Final product concentration [g/L]**	**Volumetric productivity [g/L/h]**	**Specific productivity [mg**_**product**_ **/g**_**biomass**_ **/h]**	**Yield [%]**	**Conversion [%]**
0.4	29.9 ± 0.1	3.57 × 10^8^ ± 1.15 × 10^8^	37.8 ± 2.3	0.56 ± 0.03	20.2 ± 1.3	77.8 ± 4.8	99.9 ± 0.1
0.5	37.7 ± 2.3	2.49 × 10^8^ ± 3.97 × 10^7^	26.4 ± 1.4	0.39 ± 0.02	11.2 ± 1.3	62.6 ± 3.5	100 ± 0
0.75	72.8 ± 6.2	6.28 × 10^8^ ± 1.32 × 10^8^	19.2 ± 2.1	0.28 ± 0.03	4.2 ± 0.8	32.2 ± 3.7	100 ± 0
1.25	118.3 ± 1.3	7.86 × 10^8^ ± 1.56 × 10^8^	1.2 ± 0.1	0.02 ± 0.00	0.2 ± 0.0	2.3 ± 0.3	99.3 ± 0.1

Data represent the mean (±standard deviation) of independent experiments performed in duplicate using DASGIP 8 × 1 L parallel bioreactor system as described in methods.

#### Storage of substrate in lipid bodies led to decreased product yield

Neutral lipids, such as triacylglycerols (TAGs) and sterol esters (SEs), can be stored in large quantities by oleaginous microorganisms in the form of lipid bodies (LBs) surrounded by a monolayer of phospholipids (PLs)^18^. Since *C. tropicalis* is described as an oleaginous yeast^19,20^, we speculated that the observed intracellular inclusions were LBs (Fig. 3). To confirm this, total lipids were extracted from cells under high (1.25 g/h) and low (0.4 g/h) glucose feed rates. Cells exposed to higher glucose feed rate were found to contain a twofold greater amount of lipids (183.6 ± 9.6 mg per gram of cell wet weight), compared to the cells exposed to a lower glucose feed rate (Supplementary Fig. S3). Significantly, 1.25 g/h glucose feed rate led to a fourfold higher cell dry weight but only a twofold increase in viable cells (CFU) compared 0.4 g/h (Table 2), suggesting that LB formation within yeast cells increased overall lipid content and therefore the cell dry weight.

**Figure 4:**
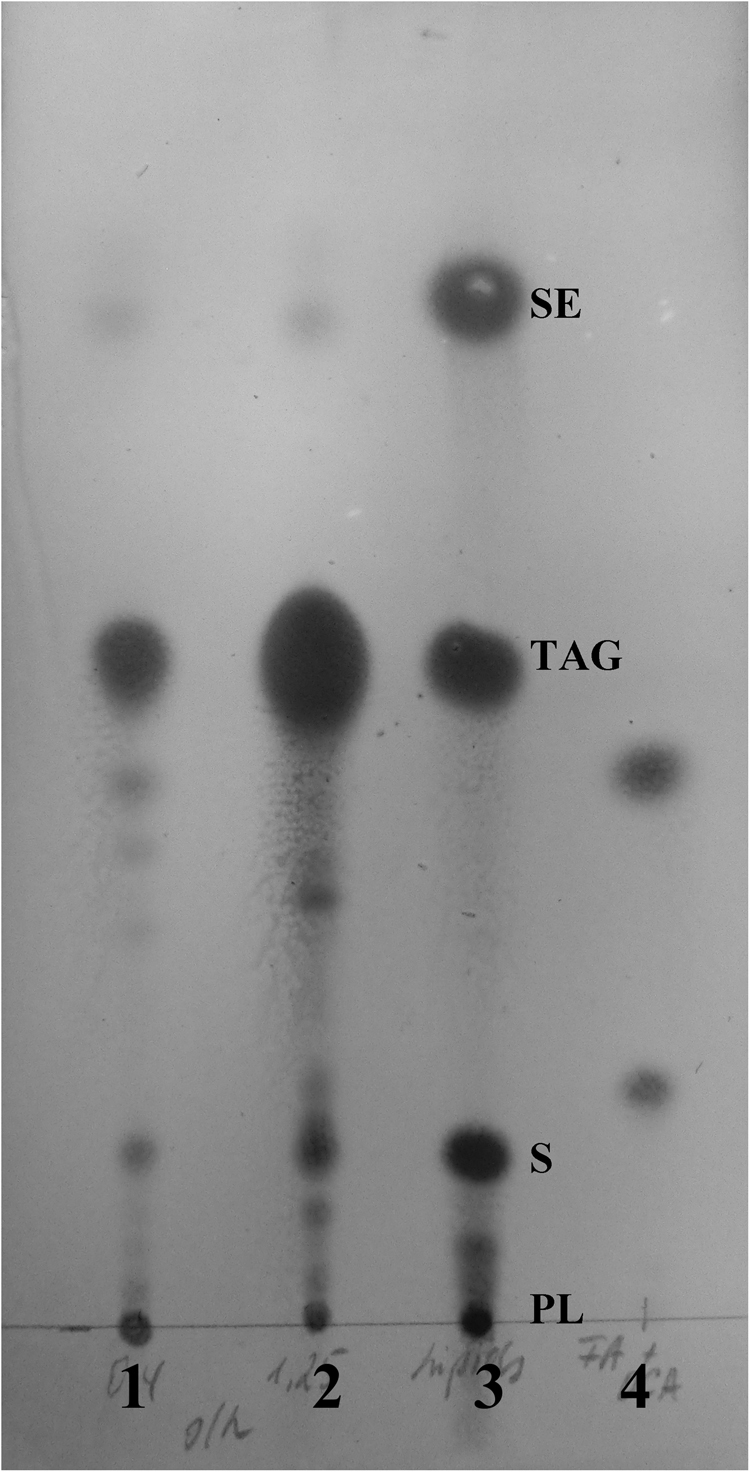
Thin layer chromatography of lipids (PLs, S, TAGs, SEs) obtained from samples exposed to (1) 0.5 g/h glucose feed rate or (2) 1.25 g/h glucose feed rate, (3) standards at 4 mg/µL and (4) oleic acid (top) and 1,18-*cis*-octadec-9-enedioic acid (bottom) at 25 mM. Figure was edited as described in methods, for original figure refer to Supplementary Information.

The composition of extracted lipids was further analysed by TLC. Extracted lipids from cells exposed to both low and high glucose feed rates were composed of TAGs, SEs, PLs and sterols (Ss) in various amounts (Fig. 4). The sterol ester level was moderate while TAGs accounted for the major fraction independent of the glucose feed rate. Similarly, stimulation of TAG formation by oleic acid with a concomitant SE level decrease was reported for *S. cerevisiae* ^21^. Thus, TAGs were likely the major LB components in *C. tropicalis* as observed in our study.

Exposure of *C. tropicalis* to higher glucose feed rate conferred a marked increase in TAG level with a simultaneous decrease in product yield (Fig. 4), indicating possible storage of oleic acid in the form of TAGs. To investigate the FA composition of TAGs, saponification of extracted lipids was performed what led to release of FAs, which were identified to be mainly oleic acids (80%, based on the peak area) as measured by GC/FID (Supplementary Fig. S4). However, no *cis*-ODA was detected. Hydrolysis of lipid extracts also led to release of glycerol, which was identified using HPLC (Supplementary Fig. S5). While saponification of crude lipid extract and subsequent HPLC and GC/FID analyses alone cannot provide information on the sources (TAGs or PLs) of released oleic acid, combined HPLC, GC/FID and TLC analyses of cells strongly suggested that yeast incorporated oleic acid mainly in the form of TAG into LBs under high glucose feed conditions, resulting in low product yield. This result highlights the importance of glucose feed rate as a critical optimisation parameter.

However, even at the lowest glucose feed rate of 0.4 g/h, a product yield of only 77.8% with simultaneous 100% conversion was achieved (Table 2). Further, the total lipid amount was fivefold higher compared to cells grown in the absence of oleic acid (Supplementary Fig. S3), indicating the formation of LBs also at lower glucose feed rates. In a previous study, *C. tropicalis* was shown to produce LBs accounting up to 58% of dry biomass when grown on glucose under nitrogen limitation^19^. However, to the best of our knowledge, this is the first description of LB formation by *C. tropicalis* during biotransformation with simultaneous glucose and oleic acid feeding.

#### Glucose excess as the main reason for oleic acid incorporation into LBs

Production of LBs by oleaginous yeasts is a well described process. The most favourable conditions for lipid accumulation is nitrogen source limitation induced by increase of molar ratio of glucose to nitrogen (C/N) greater than 20^19,20^. The C/N molar ratio at the beginning of the growth phase in this study was 8, which is theoretically the highest ratio possible during the whole process. Unlike glucose, there was no exogenous nitrogen source during the process that could finally lead to an increased C/N ratio. However, low product yield with simultaneous full substrate conversion was observed at the beginning of the biotransformation phase, indicating rapid formation of LBs (Supplementary Fig. S2) under conditions where no nitrogen limitation is expected. Interestingly, accumulation of LBs in *Yarrowia lipolytica* was also found to be independent of the ammonium concentration when hydrophobic substrates (mainly FAs) were supplied as the carbon source^22,23^.

**Figure 5:**
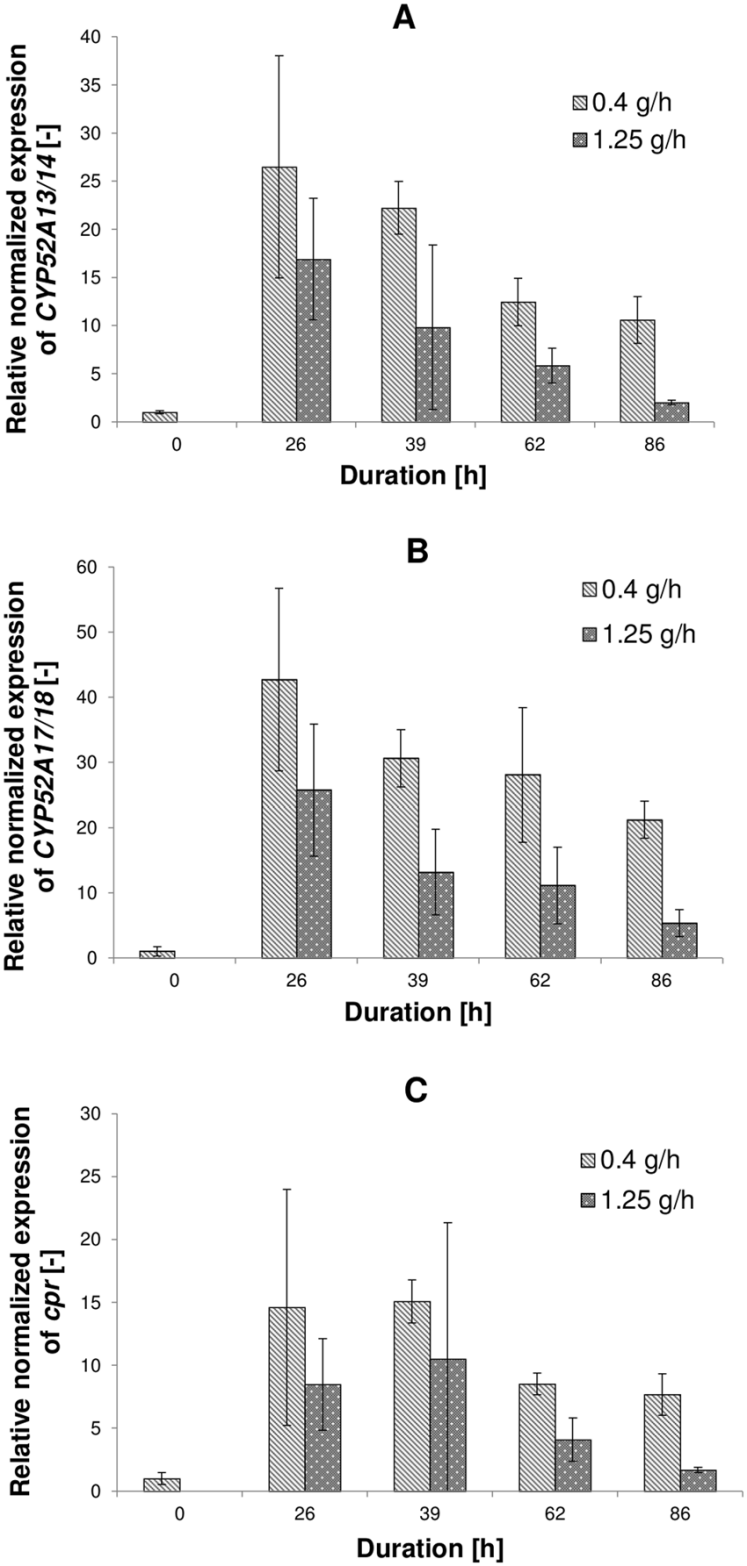
Comparison of the gene expression profiles for (**A**) *CYP52A13/14*, (**B**) *CYP52A17/18* and (**C**) *cpr* at different glucose feed rates. All expression values were normalised to *ACT1* and *GAPDH* (CV = 0.3333, M = 0.9626). The error bars represent the standard error of the mean of two biological samples measured in triplicate. Gene expression: p-value < 0.05 (except for the following sample of *cpr* expression: 1.25 g/h for 86 h).

Thus, the glucose feed rate represents the only difference between experiments and thus the main reason for reduced *cis*-ODA production. Notably, neither glucose nor ethanol accumulation in the media was observed at any glucose feed rate tested. To investigate the role of glucose in more detail, expression levels of genes encoding cytochrome P450 monooxygenase (CYP) and an associated cytochrome P450 reductase (CPR), which catalyses the first and rate-limiting conversion step of FAs to dicarboxylic acids, were analysed. As described previously, allelic variants *CYP52A13*/14 and *CYP52A17*/18 were found to be strongly induced by long-chain FAs^24,25^ and therefore were included in the investigation. Transcriptional profiles of these genes were monitored through the whole process at different glucose feed rates using RT-qPCR. The results revealed a two- to threefold decrease in the expression levels of all investigated genes towards the end of the process at the highest glucose feed rate (1.25 g/h) compared to the lowest (Fig. 5). These relatively modest differences in gene expression levels indicate only partial repression of *CYP52A* at the transcriptional level by glucose in the presence of oleic acid. In contrast, Segezzi *et al*. reported complete repression of *CYP52A* family genes by glucose; however, no information on pre-induction or simultaneous supply of FAs was provided so direct comparison with the current study is not possible^13^. Since, the observed decline of *CYP* expression at high glucose feed rate is insufficient to account for the complete inhibition of *cis*-ODA production, inhibitory effects of glucose on enzymes involved in dicarboxylic acid production are likely, as suggested in other studies of *Candida sp*.^2,14^.

**Figure 6:**
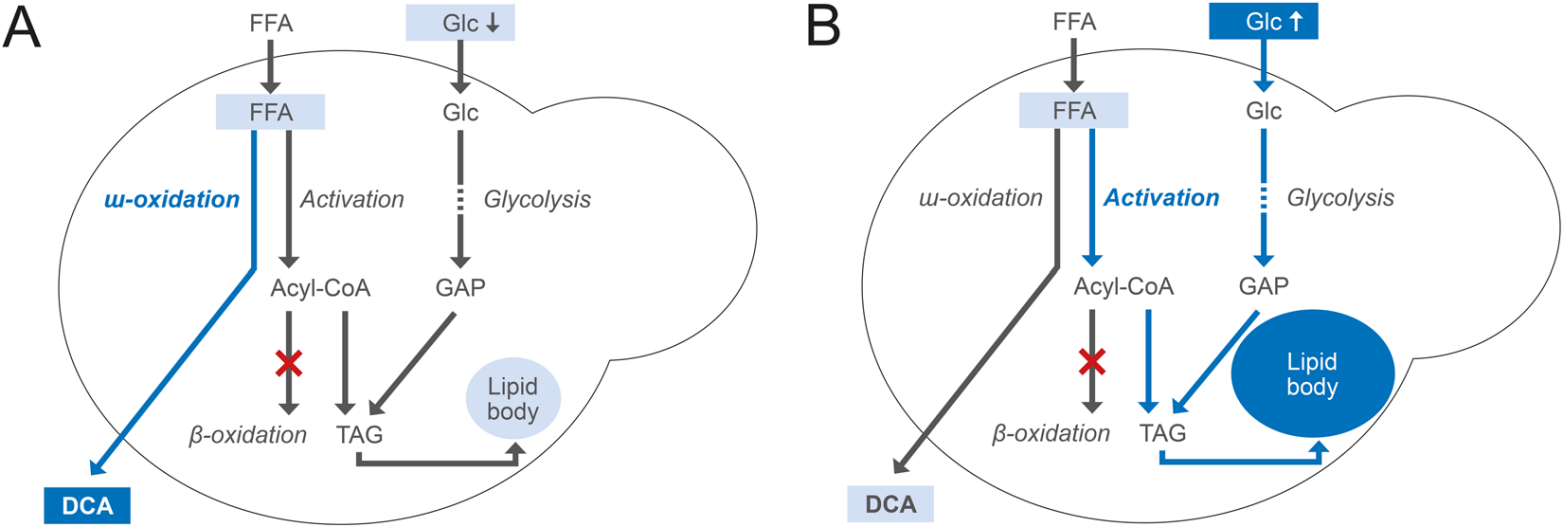
A graphical abstract: Schematic overview of oleic acid incorporation into lipid bodies (LBs). (**A**) At low glucose levels, fatty acids (FAs) are mostly converted to dicarboxylic acids through the ω-oxidation pathway and secreted into the media. (**B**) High glucose levels lead to accumulation of free FAs within the cell, resulting in TAGs formation and their storage in LBs. Glc: glucose, GAP: glycerol-3-P, FFA: free fatty acid, TAG: triacylglycerol.

Since, the biogenesis of TAGs is driven by the availability of precursors needed for synthesis, namely activated FAs and either glycerol-3-phosphate or dihydroxyacetone derived from glycolysis^18^, we assumed that glucose excess suppresses ω-oxidation at the transcriptional or enzymatic level, resulting in elevated oleic acid and greater TAG production (Fig. 6). Similarly, *Y. lipolytica* was reported to produce large LBs when the degradation of FAs (β-oxidation) in peroxisomes was interrupted and glucose was channelled toward TAG production by knockout of *pox* and *gut2* genes, respectively^22^. Thus, the supply of oleic acid in combination with excessive glucose likely leads to the incorporation of oleic acid in LBs as TAGs, resulting in a massive decrease in product yield.

#### Influence of substrate chain length on lipid body formation and substrate toxicity

To our knowledge, formation of LBs by incorporation of FAs supplied as a substrate during dicarboxylic acid production has not been described. Also, in one of our previous studies on production of dodecanedioic acid (DDA), no accumulation of fatty acid methyl ester as a substrate (dodecanoic acid methyl ester (DAME)) or corresponding fatty acid (dodecanoic acid, DA) in LBs was observed, although a relatively high glucose feed rate of 1 g/h was applied^17^. In comparison, a glucose feed rate of 0.5 g/h was sufficient to facilitate LB formation using oleic acid as a substrate. These observations suggest either that the glucose feed rate of 1 g/h may have still been too low to initiate LB formation or that C12 FAs are not suitable for LB production. To distinguish these possibilities, the production process was repeated with DAME as substrate at two different glucose feed rates, 1 g/h and 3 g/h, under optimised conditions, including the pH shift in the growth phase. Interestingly, no LB formation was observed using DAME as a substrate at high glucose feed rate as evidenced by microscopic monitoring of cells and by the comprehensive mass balance of the substrate and the product (Supplemental Fig. S6). Similarly, Froissard *et al*. found that incorporation of medium-chain fatty acids (MCFAs) into LBs occurred very rarely, accounting for only 0.5% of total FA content^26^. Alternatively, MCFAs can be stored in LBs after elongation^27^, but due to a comprehensive mass balance, any further utilisation of DA in our study can be excluded. In contrast, recent research by Mishra *et al*. suggested the channelling of DA as an intermediate in DDA production from *n*-dodecane into a *de novo* FA pathway based on an established *in silico* model using *C. tropicalis* ATCC 20962^11^. However, no further information was provided on subsequent utilisation, including LB formation from elongated FAs.

Under identical conditions, DAME was found to be much more toxic to *C. tropicalis* than oleic acid. Thus, the pre-induction of cells with DAME (0.2% v/v) at the beginning of the growth phase led to an almost fourfold decrease in biomass (6.1 ± 0.8 g/L, n = 4) compared to the trials induced by oleic acid (Table 1). As Green *et al*. suggested, dicarboxylic acid production reflects a balance between FA toxicity and nutritional value^14^. Since the applied *C. tropicalis* strain is not able to utilise FAs as a carbon source, dicarboxylic acids production remains the main way to avoid lipotoxicity. However, LB formation may also represent an optional detoxification pathway by storing FAs in TAGs. Thus, oleic acid, which was found to be incorporated into LBs, appears to have no toxic effects on the biological activity of *C. tropicalis*. In contrast, DAME as a MCFA is not stored in LBs, resulting in high toxicity.

### Oleic acid feed rate influenced *cis*-ODA production yield

While glucose excess was identified as responsible for oleic acid incorporation into LBs, oleic acid itself as a second important precursor of TAGs may also influence *cis*-ODA yield (Fig. 6). To minimise the concentration of oleic acid available for TAG production, an optimal oleic acid feed rate was investigated at a constant glucose feed rate of 0.4 g/h.

Surprisingly, low oleic acid feed rate (0.3 g/L/h) resulted in low productivity, while increasing the oleic acid feed rate enhanced product yield, reaching 60.6 ± 2.5% at 1.0 g/L/h (Supplementary Fig. S7). Thus, oleic acid induction appears to be competitive with glucose suppression of ω-oxidation, and a certain molar ratio of glucose to oleic acid (C/S) is required to initiate LB formation. Since the glucose feed was kept constant throughout the whole biotransformation phase, increased oleic acid feed rate decreased the C/S ratio, resulting in enhanced *cis*-ODA production. However, reducing the glucose feed rate below 0.4 g/h led to an immediate loss of cell viability (as indicated by a rise in dissolved oxygen and foaming). Nevertheless, product yield of 70% was possible at the lowest possible molar ratio of 1.5.

A loss of ~30% oleic acid to LB formation appears inevitable under the conditions applied in this study. To investigate possible methods for degradation of LBs, the glucose feed was interrupted and the levels of oleic acid and *cis*-ODA were monitored. Under these conditions, however, release of neither oleic acid nor *cis*-ODA was observed. The degradation of TAGs can be activated by transferring the cells into fresh media containing glucose^18^, but this procedure would not be efficient for industrial production. A knockout of the major regulator of TAG synthesis, phosphohydrolase, leading to a 70% reduction of TAG^18^ production may be a more promising method.

Nevertheless, using an oleic acid feed rate of 1 g/L/h and a glucose feed rate of 0.4 g/h for a given biomass of 33 g/L yielded a product concentration of 42.0 ± 5.7 g/L (Table 3). The volumetric productivity of 0.56 g/L/h obtained in this study is the highest described so far for *cis*-ODA production by *C. tropicalis* ATCC 20962. For instance, Zibek *et al*. and Yang *et al*. reported lower volumetric productivities of 0.4 g/L/h and 0.43 g/L/h, respectively^8,9^. Genetic engineering approaches can improve productivity immensely. Piccatagio *et al*. reported a volumetric productivity of 1.5 g/L/h by the overexpression of cytochrome P450 monooxygenase (CYP) and associated cytochrome P450 reductase in *C. tropicalis* ^1^. In contrast to our study, however, none of these aforementioned studies provided any evidence of LB formation during FA biotransformation. Thus, our study demonstrates for the first time the incorporation of oleic acid into LBs during *cis*-ODA production.

**Table 3:** Parameters calculated for optimal conditions (glucose feed at 0.4 g/h and oleic acid feed at 1 g/L/h).

Final cell biomass [g/L]	Colony forming units [CFU/mL]	Final product concentration [g/L]	Volumetric productivity [g/L/h]	Specific productivity [mg_product_/g_biomass_/h]	Yield [%]	Conversion [%]
33.3 ± 4.9	3.7 × 10^8^ ± 2.7 × 10^7^	42.0 ± 5.7	0.56 ± 0.03	16.5 ± 3.8	72.8 ± 4.9	99.9 ± 0.6

Data represent the mean ± standard deviation of six independent experiments performed using the DASGIP 8 × 1 L parallel bioreactor system as described in the methods.

To summarize, this study describes the development of a highly efficient *cis*-ODA production process by whole-cell biotransformation using *C. tropicalis* in a bench-top parallel bioreactor system. The optimisation involved modification of both growth and biotransformation phases. The growth phase was optimised to yield larger amounts of biocatalyst and pH modulation for enhanced substrate and product solubility. *cis*-ODA production in the biotransformation phase was strongly influenced by glucose and oleic acid feed rates. We have also shown for the first time that elevated glucose feed rate leads to low biotransformation efficiency due to the incorporation of oleic acid into triacylglycerols for storage in LBs. To channel oleic acid into ω-oxidation, a low molar ratio of glucose to oleic acid was necessary. Even though the lowest possible molar ratio of glucose to oleic acid (1.5) resulted in some lipid body formation, a 70% product yield was achieved and a comparably high volumetric productivity of 0.56 g/L/h by *C. tropicalis* ATCC 20962 was obtained.

## Methods

### Materials

Oleic acid (technical grade, 94.7%, Alfa Aesar, Germany) was used as the starting material for biotransformation. Analytical standard oleic acid (≥99% by gas chromatography) was purchased from Sigma Aldrich (Germany). Analytical standard *cis*-ODA was produced and purified as described in the Supplementary Information.

### Microorganisms

The *Candida tropicalis* ATCC 20962 yeast strain (Genotype: *pox5:ura3A pox5:ura3A pox4A:ura3A pox4B:URA3A*) with a blocked β-oxidation pathway was purchased from the American Type Culture Collection (ATCC).

### Cultivation conditions for preculture in shaker flasks

A single *C. tropicalis* colony cultivated on YPD agar plates (10 g/L yeast extract, 20 g/L peptone, 20 g/L glucose, 15 g/L agar-agar) for 48 h at 30 °C was transferred into 15 mL YPD liquid medium (10 g/L yeast extract, 20 g/L peptone and 20 g/L glucose) and cultivated for 12 h at 30 °C with 150 rpm shaking. The obtained cell broth was used for the inoculation of 150 mL YPD medium at OD_600_ ∼1 and cultivated for an additional 12 h at 30 °C with 150 rpm shaking.

### Cultivation conditions for preculture in a 2-L bioreactor

A 2-L bioreactor (Sartorius AG, Germany) was used to preculture *C. tropicalis* cells for the whole-cell biotransformation process in the DASGIP bioreactor system (Eppendorf AG, Germany). The 2-L bioreactor was inoculated by adding 100 mL cell suspension from the shaker flask into 900 mL OPT-1 medium (30 g/L glucose, 8 g/L (NH_4_)_2_SO_4_, 1 g/L K_2_HPO_4_, 2 g/L KH_2_PO_4_, 4.5 g/L yeast extract, 0.1 g/L NaCl, 0.1 g/L CaCl_2_, 4 mM MgSO_4_ and 1 mL/L trace elements solution containing 0.4 g/L MnSO_4_·H_2_O, 0.4 g/L ZnSO_4_·7H_2_O, 0.1 g/L KI, 0.5 g/L H_3_BO_3_, 0.6 g/L FeCl_3_·6H_2_O and 0.04 g/L CuSO_4_·5H_2_O). Cultivation was performed at 30 °C and pH 5.8, with aeration at 0.24 L/min and agitation at 600 rpm. After 8 h of cell growth, additional glucose was supplied at a final concentration of 30 g/L and cultivation continued for another 4 h.

### Whole-cell biotransformation in DASGIP

The whole-cell biotransformation of oleic acid was performed using an 8 × 1 L parallel bioreactor system (DASGIP). The bioreactors were inoculated by adding 30 mL yeast cell suspension produced in the 2-L bioreactor into 250 mL OPT-1 medium (final volume of 280 mL), followed by induction with 0.5 mL oleic acid or dodecanoic acid methyl ester (DAME). During the growth phase, pH was maintained at 5.8 by the addition of 6 N NaOH. After 8 h of cultivation, additional glucose was supplied at a feed rate of 8 g/h for 1 h (final added concentration of 30 g/L). At the same time, pH was shifted from 5.8 to 8.0 within 6 h. The biotransformation was conducted after the growth phase (16 h) by initiation of glucose and substrate feed at various rates. Glucose feed rate is given in g/h and is independent of bioreactor volume. Whereas oleic acid feed is related to the current volume in bioreactor and is given in g/L/h. During biotransformation, pH was maintained at 8.0 by addition of 6 N NaOH. The entire process was performed at 30 °C. Aeration was set to 6 sL/h and dissolved oxygen concentration was maintained at 15% by varying the agitation speed (600–1,200 rpm). Antifoam agent was added if needed.

### Sampling and samples storage

During the production process, samples were continuously taken for analyses. A schematic overview of sample treatment and storages conditions is presented in Supplementary Fig. S8.

### Microscopy

Microscopic images were obtained using a Motic Microscope BA310 and Motic Images Plus 2.0 software. ImageJ 1.51d software and an object measuring plate (Mikroskop Technik Rathenow, Germany) were used for image analysis (cropping, splitting color channels and insertion of size bar).

### Colony forming units (CFUs)

Colony forming units were determined by seeding samples at different dilutions on YPD agar plates. After incubation at 30 °C for 24 h, the colonies were counted.

### Cell dry weight determination

For determining the cell dry mass, 1 mL fermentation broth was centrifuged (21,000 × *g*, 5 min, room temperature (RT)). The supernatant was removed for glucose assay and thin layer chromatography, while the cell pellet was completely dried (24 h) at 65 °C and weighed.

### Thin layer chromatography (TLC)

A 3 µl supernatant sample was spotted on a TLC plate (TLC Silica gel 60 F254, Merck) and separated using *n*-Hexane:diethyl ether:acetic acid (80:20:2 v/v) as the mobile phase. Spots were visualised using phosphomolybdic acid dissolved in ethanol (48 g/L). To verify the composition of extracted lipids, the following standards were used: dioleoyl-sn-glycero-3-phosphocholine, ergosterol, triolein and cholesteryl oleate. Obtained figures were edited by appropriate cropping, labelling the lines and splitting color channels using ImageJ 1.51d software.

### Glucose assay

A photometric enzyme assay was applied to measure supernatant glucose concentration as described earlier^28^. Briefly, supernatant samples were mixed 1:1 with assay mixture solution (40 mM potassium phosphate, pH 6.0, 1.5 mM 2,2-azino-bis-(3-ethylbenzthiazoline)-6-sulfonic acid, 0.4 U glucose oxidase and 0.02 U horseradish peroxidase) and incubated at 30 °C for 30 min with 400 rpm shaking. The resulting extinction at 418 nm was measured and subtracted from absorption at 480 nm to eliminate background signals.

### Extraction and quantitative analysis of the product by gas chromatography with flame ionisation detection (GC/FID)

Samples from the cultivation broth were acidified using 2 N HCl and extracted with two volumes of methyl-tert-butyl ether on a rocking shaker for 2 h. For GC/FID measurements, four volumes of the organic supernatant were mixed with one volume of N-methyl-N-(trimethylsilyl) trifluoroacetamide for silylation of the hydroxyl groups. Samples were measured along with appropriate amounts of standards using a gas chromatograph (ModelTrace GC Ultra, Thermo Fischer Scientific) equipped with an autosampler (Model TriPlus), flame ionisation detector and Rxi®-5Sil MS column (Restek, 30 m length, 0.25 mm I.D., 0.25 µm film thickness). The He carrier gas flow was set to 0.5 mL/min and held at 90 °C for 3.5 min, ramped to 210 °C at 50 °C/min, ramped to 220 °C at 10 °C/min, ramped to 280 °C at 15 °C/min, ramped to 330 °C at 60 °C/min and held at 300 °C for 1.5 min.

### High-performance liquid chromatography (HPLC)

Ethanol and glycerol in aqueous phase, released after the saponification of lipids was quantified by HPLC as described^29^. Briefly, samples were analysed using a HPLC system (Dionex^®^, Sunnyvale, CA, USA) equipped with a Rezex ROA-H^+^ column (Phenomenex^®^, Torrance, CA, USA), a refractive index detector (RI 101, Shodex, Tokyo, Japan) and a PDA detector (210/278 nm, Dionex^®^, Sunnyvale, CA, USA). The mobile phase (sulfuric acid, 2.5 mM) flow was set to 0.5 mL/min at an oven temperature of 70 °C.

### RNA isolation, reverse transcription and RT-qPCR

The yeast cells were first pretreated with zymolyase (Longlife™ Zymolyase® G-Biosciences, USA) and RNA extracted using the Aurum™ Total RNA Mini Kit (Bio-Rad, USA) according to the manufacturer´s protocol. An additional gDNA degradation step was performed using the RapidOut DNA Removal Kit (Thermo Fisher Scientific, USA), followed by reverse transcription of 160 ng of total RNA using the iScript cDNA synthesis kit (Bio-Rad, USA). Quantitative PCR was conducted using SsoAdvanced Universal SYBR Green mixture (Bio-Rad, USA) and a CFX96 Touch™ Real-Time PCR Detection System (Bio-Rad, USA) with the following thermocycle conditions: 95 °C for 30 s, followed by 40 cycles of 95 °C for 15 s and 61 °C for 15 s and a final step at 95 °C for 10 s. For each PCR product, the dissociation protocol was applied as followed: melting curve from 65 °C to 95 °C in 0.5 °C increments every 5 s. Additionally, PCR products were analysed on 2% agarose gels. PCR efficiency was determined for each primer pair according to the following equation: E(%) = (10^(−1/slope)^ − 1) × 100. Sequences of primer pairs and accession numbers of target genes are listed in Supplementary Table S1. Technical variability between samples was minimised using an inter-run calibrator. Expression data were analysed using Bio-Rad CFX Manager (Bio-Rad, USA) following the instructions of geNorm^30^. *GAPDH* and *actin* were used for normalisation (expression stability, M = 0.9626, and coefficient of variation, CV = 0.3333).

### Total lipid extraction and saponification

Total lipid was extracted from yeast using a chloroform:methanol:water mixture as described^31^. Briefly, approximately 0.1 g cell pellet was obtained by centrifugation (5 min, 21,000 × *g*, RT), dissolved in 1.5 mL methanol:chloroform (2:1, v/v) and vortexed for 6 min with glass beads (∅ = 1 mm). Then, 500 µL chloroform was added and the mixture vortexed for another 1.5 min. This step was then repeated after adding 500 µL ddH_2_O. After phase separation, the lower chloroform phase containing extracted lipids was recovered and the chloroform evaporated. Prior to saponification, lipids were solubilised in 1 M KOH (in 70% ethanol) and heated in a water bath at 70 °C–75 °C for 1 h. The lipid mixture was acidified with sulfuric acid, and released FAs were extracted using *n*-hexane and analysed using GC/FID. The glycerol remaining in the aqueous phase was analysed using HPLC.

### Data availability

The datasets generated during the current study are available from the corresponding author on reasonable request.

## Electronic supplementary material

Supplementary Information
