# Supplementary material for: Effects of glucose concentration on 1,18-cis-octadec-9-enedioic acid biotransformation efficiency and lipid body formation in Candida tropicalis
Source: Sci Rep. 2017 Oct 23;7:13842. doi: 10.1038/s41598-017-14173-7 (PMC5653835; doi:10.1038/s41598-017-14173-7)
Supplement: Supplementary file 1 — Supplementary Information [file 41598_2017_14173_MOESM1_ESM.doc]

Supplementary Information

**Effects of glucose concentration on 1,18-*cis*-octadec-9-enedioic acid biotransformation efficiency and lipid body formation in *Candida tropicalis***

Irina Funk^1^, Volker Sieber^1,2^ and Jochen Schmid^1*^

*^1^ Technical University of Munich, Chair of Chemistry of Biogenic Resources, Schulgasse 16, 94315 Straubing, Germany*

*^2^ Catalysis Research Center, Technical University of Munich, 85748 Garching, Germany*

* Correspondence: Jochen Schmid: j.schmid@tum

Technical University of Munich, Chair of Chemistry of Biogenic Resources, Schulgasse 16, 94315 Straubing, Germany

E-mail: j.schmid@tum.de

Tel: +49 (0) 9421 187 331

Fax: +49 (0) 9421 187 310

S1 Methods

S1 Tables

S1 Figures

Original Figures

**S1 Methods**

**Equations used for characterizing of the production process**

Specific growth rate [h^−1^] µ_max_ = (lnX-lnX_0_)/(t-t_0_) (1)

Doubling time [h] t_D_ = ln2/µ_max_ (2)

Biomass yield coefficient [g_biomass_/g_glucose_] Y_X/C_ = X/C (3)

Conversion [%] X_S_ = (S_0_-S)/S_0_ × 100% (4)

Yield [%] Y_P_ = (P-P_0_)/S_0_ × 100% (5)

Space time yield [g/L/h] STY = P/(V × t) (6)

Specific productivity [mg_product_/g_biomass_/h] Sp = P/(X_B_ × t) (7)

Reaction yield [g_product_/g_substrate_]  q_p_ = P/S (8)

Where, X- biomass (OD_600_) at the end of the growth phase, [-]; X_0_-biomass (OD_600_) at the start of the growth phase, [-]; (t-t_0_)-growth phase duration, [h]; C-glucose amount at the start of the growth phase, [g]; S_0_-amount of substrate at the start of the process, [g]; S-amount of substrate at the end of the process, [g]; P-amount of product at the end of the process, [g]; P_0_-amount of product at the start of the process, [g]; t- – process time, [h]; V -working volume at the end of the process, [L]; X_B_-cell biomass at the end of the process, [g].

**Purification of 1,18-*cis*-Octadec-9-enedioic acid**

A hot-filtration recrystallization method was used to purify 1,18-*cis*-octadec-9-enedioic acid (*cis*-ODA). First, cell broth obtained after biotransformation was centrifuged (15,000*g*, 30 min, RT). Prior to extraction, the supernatant was acidified with 2 N HCl and filtrated. The obtained solid was dissolved in a mixture of hexane and ethyl acetate (6:1), following by heating up to 90 °C for 40−60 min until boiling. The mixture was then hot-filtrated to remove insoluble impurities and then cooled to 4 °C for 12 h. The obtained crystals were filtered using a pre-cooled mixture of hexane and ethyl acetate (6:1). The obtained solid was further dried using a vacuum desiccator (12−24 h). In this way obtained *cis*-ODA with 94% purity (confirmed by GC/FID) as the analytical standard for GC/FID.

**Nuclear magnetic resonance (NMR) spectroscopy of 1,18-*cis*-Octadec-9-enedioic acid**

NMR-measurements were carried out at 25 °C using standard pulse programs on JNM-ECA 400 MHz spectrometer (JEOL, USA). Chemical shifts are given as δ-values in ppm and reported as follows: value (multiplicity, coupling constant(s) where applicable, number of protons). Coupling constants (J-values) are given in Hertz (Hz). The DEPT135° technique was used to assign CH2-signales.NMR spectra assignation was supported by comparison with literature values for similar compounds. Only clearly identifiable peaks are assigned. For the characterization of observed signal multiplicities the following abbreviations were applied: s (singlet), d (doublet), dd (double doublet), dt (double triplet), t (triplet), q (quartet), quint (quintet) and m (multiplet).

**S1 Tables**

**Supplementary Table S1**: Sequences of primer pairs used for RT-qPCR and accession numbers of target genes

| **Gene** | **Primer** | **Gene Sequence 5′-3′** | **Amplicon size**  **[bps]** | **Accession number** |
| --- | --- | --- | --- | --- |
| *ACT1* | forward | TGCTTTGGCTCCATCTTC | 147 | EER33201.1 |
|  | reverse | GGACCAGATTCGTCGTATTC |  |  |
| *GAPDH* | forward | GGTAGAACTGCTTCTGGTAAC | 101 | EER30670.1 |
|  | reverse | GACATACCAGTCAATTTACCG |  |  |
| *CYP52A13** | forward | GGTTTGAGCCAGAGACAAAG | 97 | AY230499.1 |
| *CYP52A14** | reverse | GCTTCTGTCAAGGCAAACTG |  | AY230500.1 |
| *CYP52A17** | forward | TGATGCTGCTGAGTTCAGAC | 83 | AY230504.1 |
| *CYP52A18** | reverse | CACCGTTGAATGGCAAGTAAG |  | AY230505.1 |
| *CPR* | forward | TGCCAGTAGAATGGCTAGAG | 103 | AY705446.1 |
|  | reverse | CCAGGACTTGACCAATTCAG |  |  |
|  | reverse | GTGGCAGCAGTATCATCTCTTG |  |  |

* Primers are designed for amplification for both alleles

**S1 Figures**


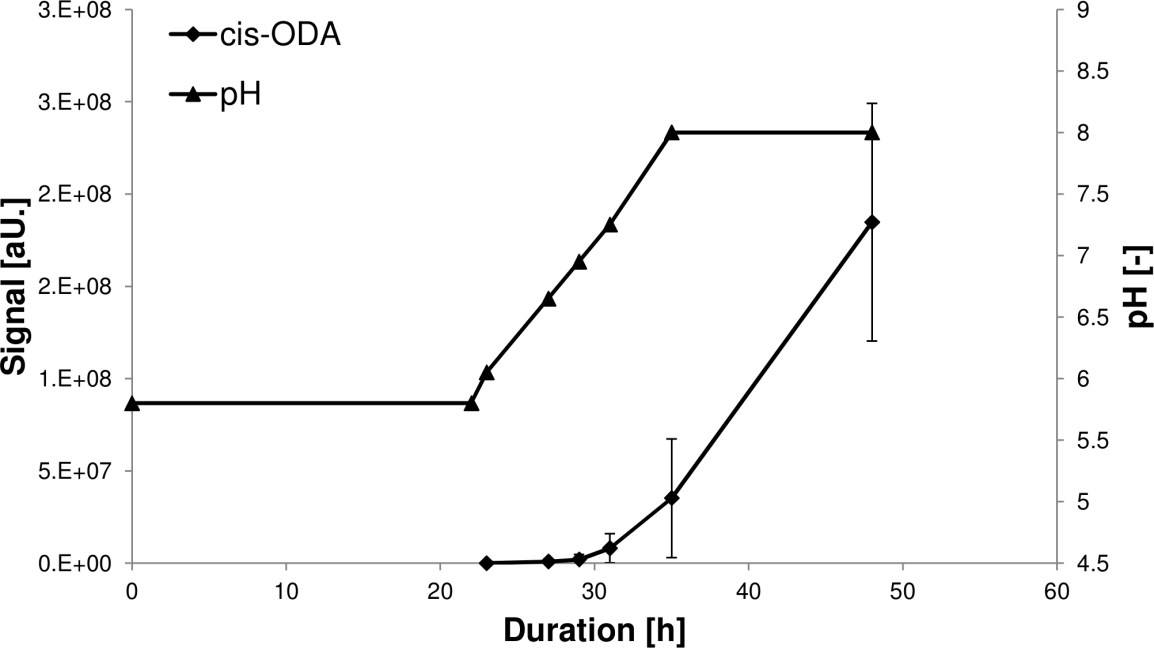


**Supplementary Figure S1**: Production of *cis*-ODA over the time versus pH shift as observed by simultaneous application of the pH shift and oleic acid feed. Enhanced production of *cis*-ODA was observed at slightly basic pH (around 8.0). The experiments were performed in duplicate using a 2 L bioreactor. The error bars represent the standard deviation.


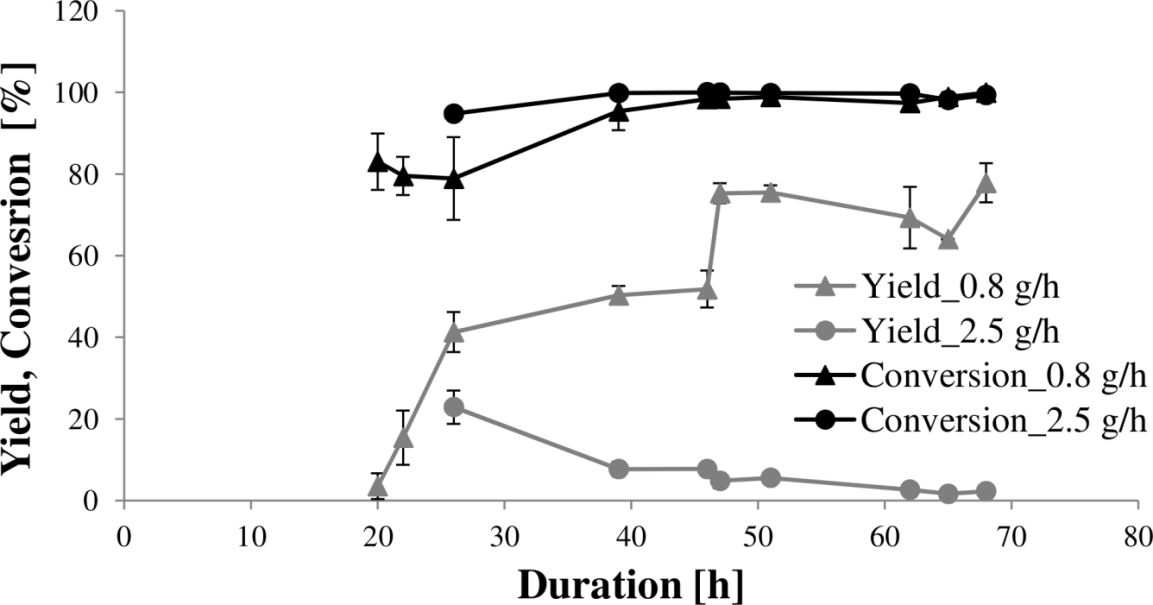


**Supplementary Figure S2:** Product yield and conversion of oleic acid over the time at different glucose feed rates during the production process. The experiments were performed in duplicate using DASGIP 8 × 1 L parallel bioreactor system. The error bars represent the standard deviation.

**
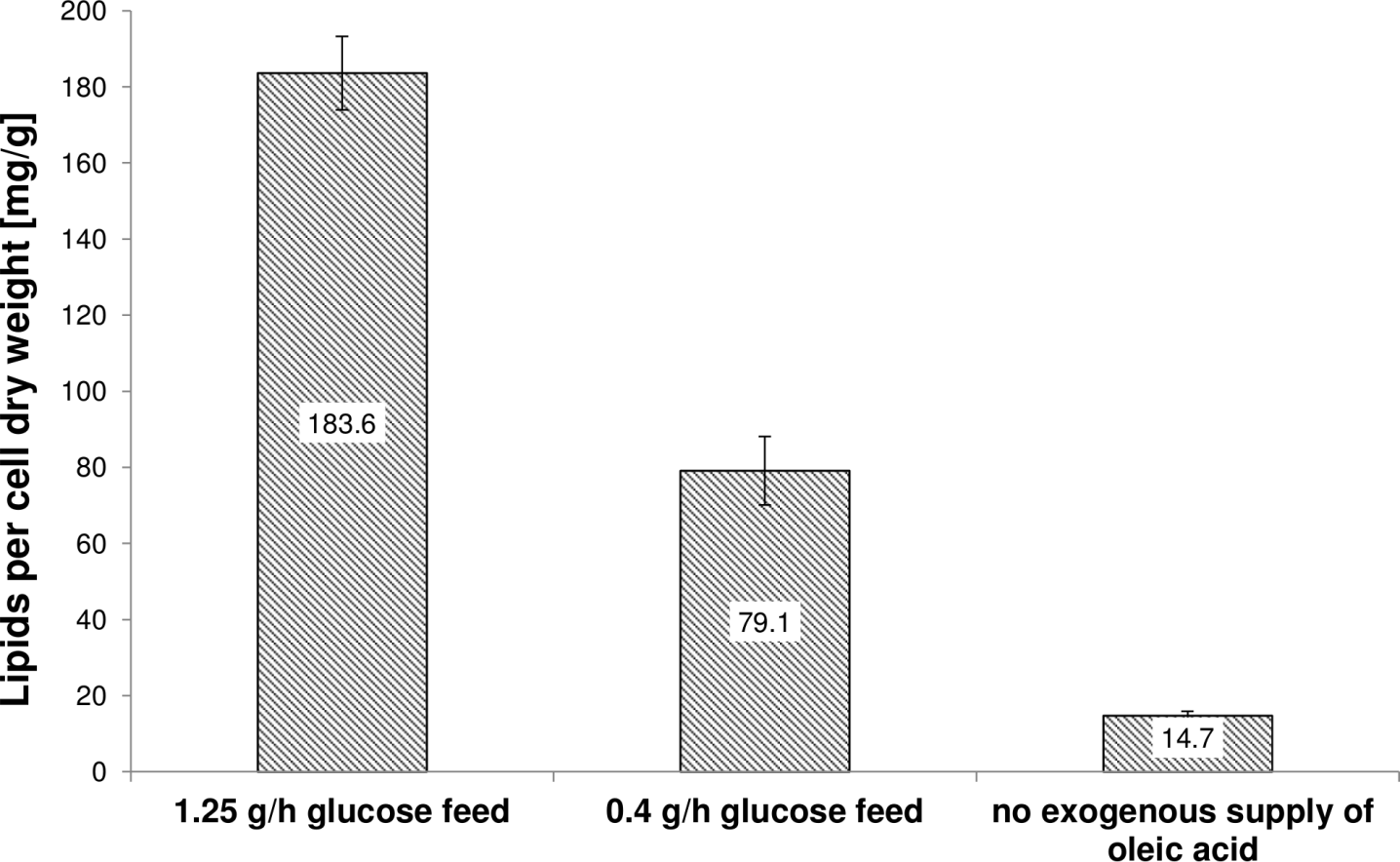
**

**Supplementary Figure S3:** Extracted total lipids from samples exposed to different glucose feeds rates. Lipid concentration is expressed per cell wet weight. Error bars represent standard deviation from three independent experiments.

**
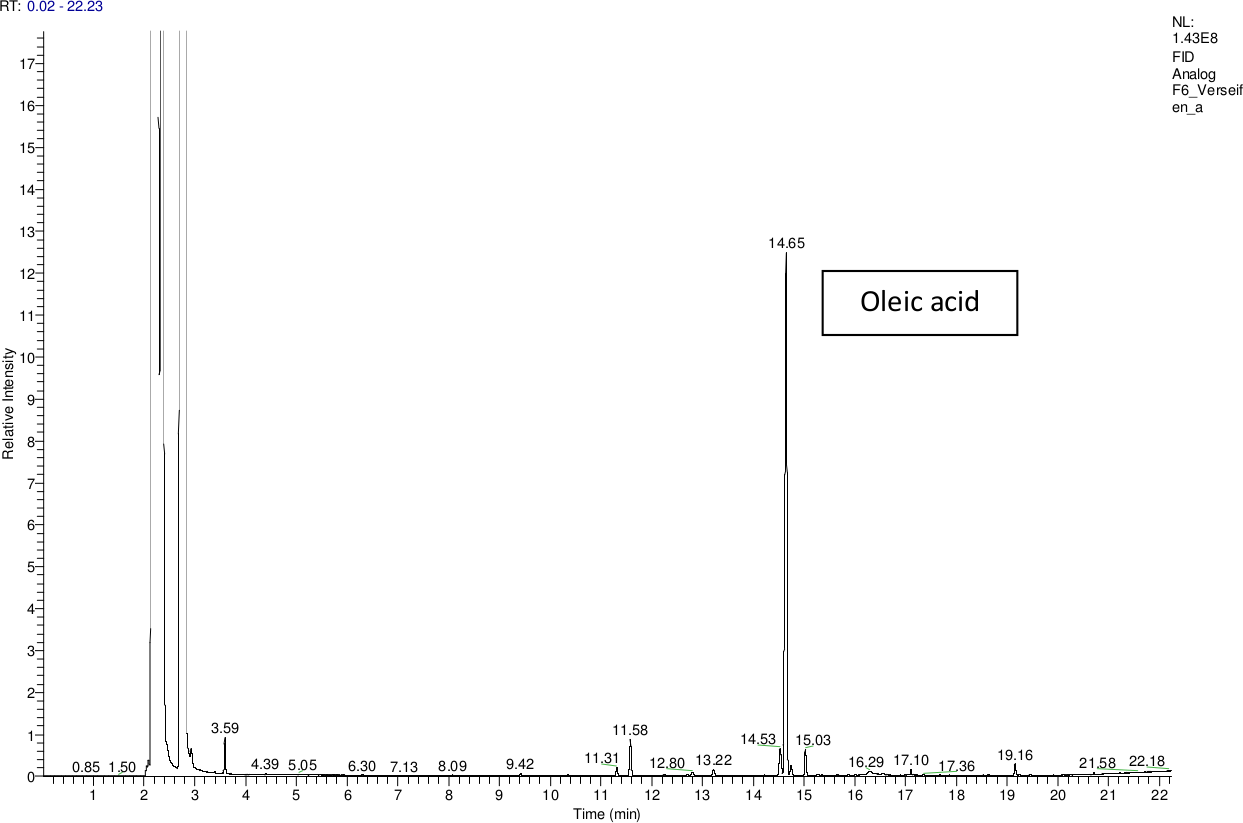
**

**Supplementary Figure S4:** GC/FID chromatogram of released fatty acids after hydrolysis of lipids. Retention times: Linoleic acid- 18.89 min, Oleic acid- 19.09 min, Stearic acid- 19.38 min.


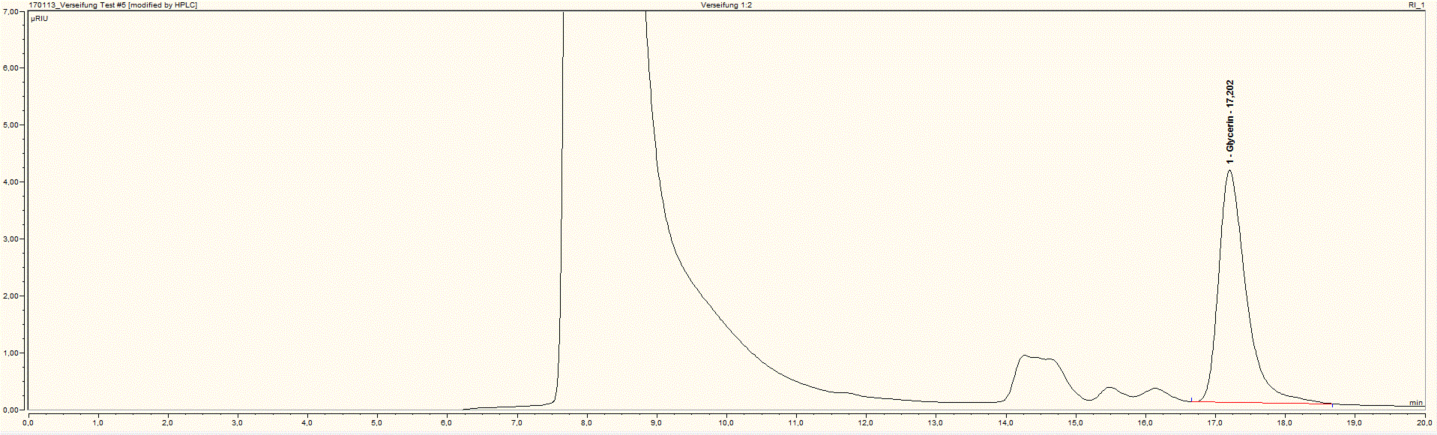


**Supplementary Figure S5:** HPLC chromatogram of released glycerol (retention time: 17.02 min) after hydrolysis of lipids.


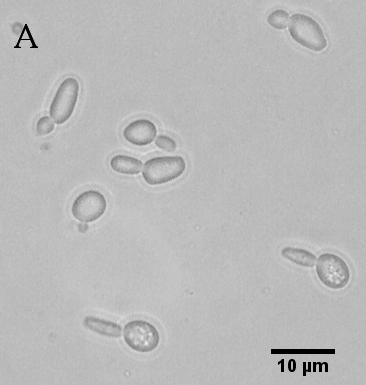

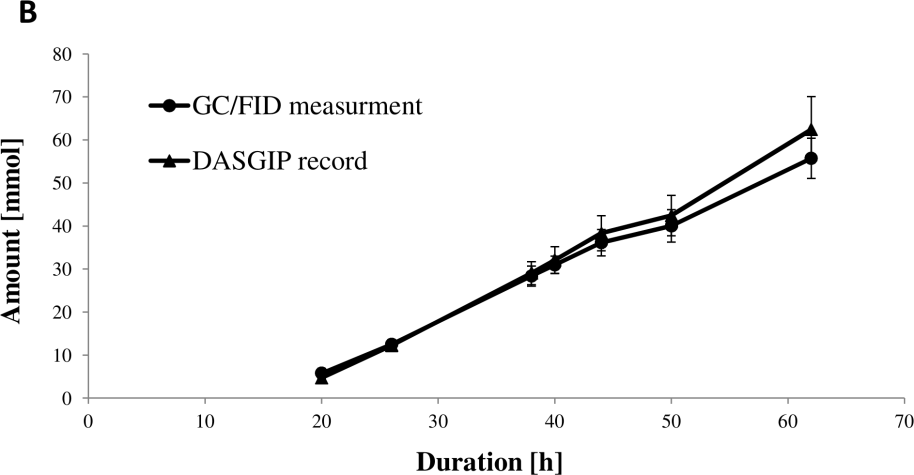


**Supplementary Figure S6:** (A) Microscopic picture at 1,000× magnification of *C. tropicalis* after 67 h of the biotransformation process using acid methyl ester (DAME) as substrate. (B) Amount of DAME as supplied and registered by calibrated DASGIP feed rates in comparison to the summarized amounts of DAME, DA, and DDA as measured by GC/FID. The experiments were performed in duplicate and the error bars represent the standard deviation.


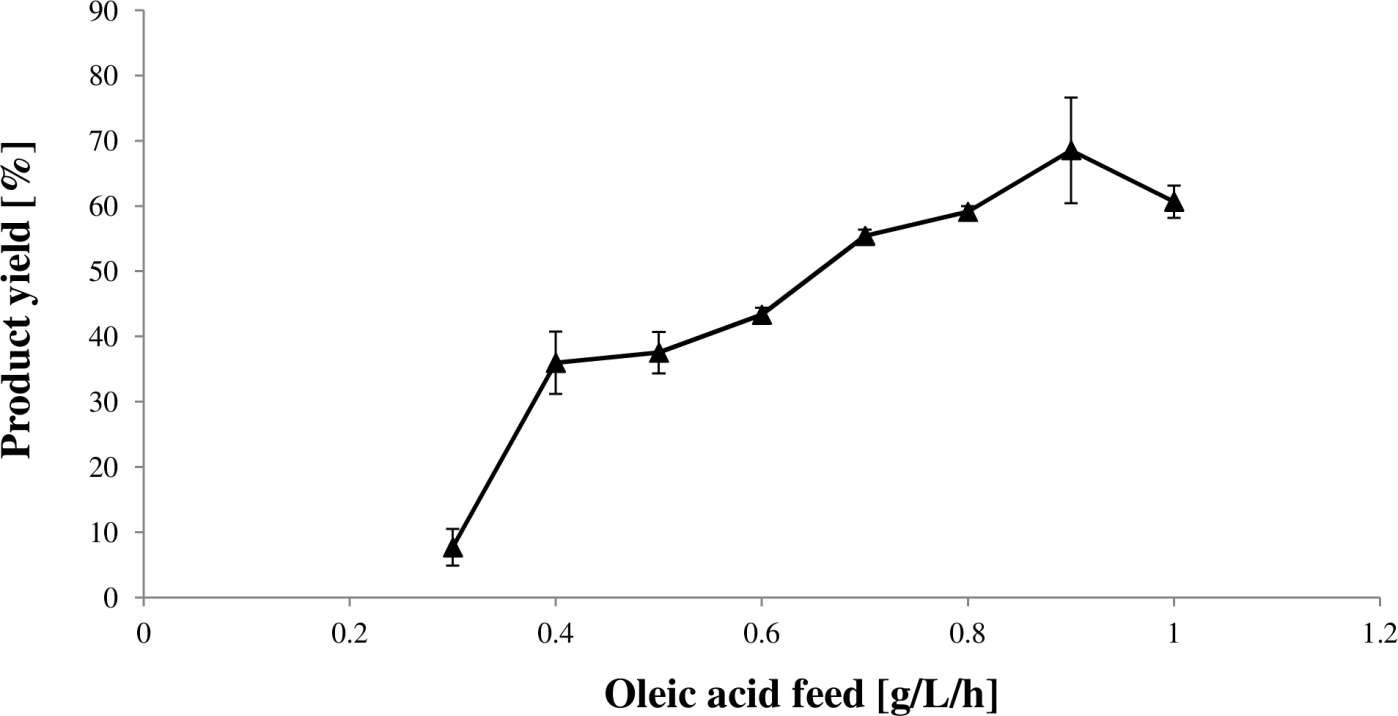


**Supplementary Figure S7:** Product yield versus oleic acid feed rate. The experiments were performed at least in duplicate using the DASGIP 8 × 1 L parallel bioreactor system as described in the methods. The error bars represent the standard deviation.

**
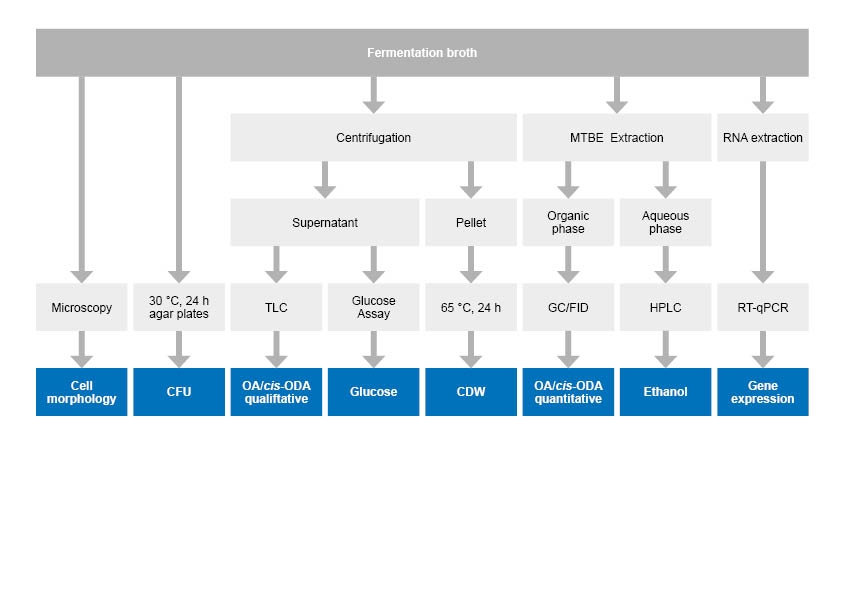
Supplementary Figure S8:** Schematic overview of the sample treatment. CFU: colony forming unit; CDW: cell dry weight; OA: oleic acid; *cis*-ODA: 1,18-*cis*-octadec-9-enedioic acid


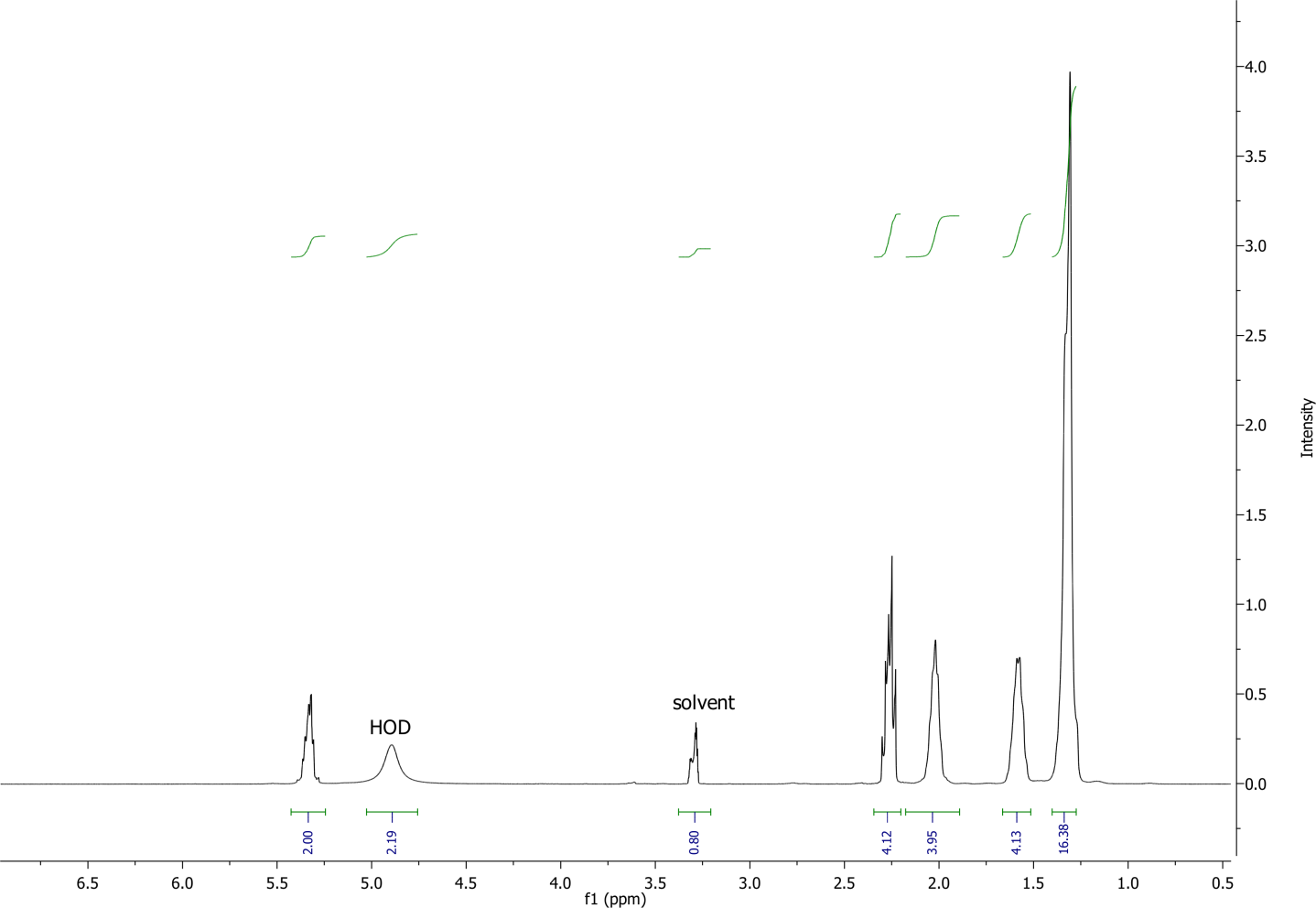


**Supplimentary Figure S9**: ^1^H-NMR of 1,18-*cis*-Octadec-9-enedioic acid (400 MHz, CD_3_OD): δ 5.41 – 5.26 (m, 1H), 2.34 – 2.21 (m, 2H), 2.09 – 1.92 (m, 2H), 1.67 – 1.50 (m, 2H), 1.31 (s, 8H).


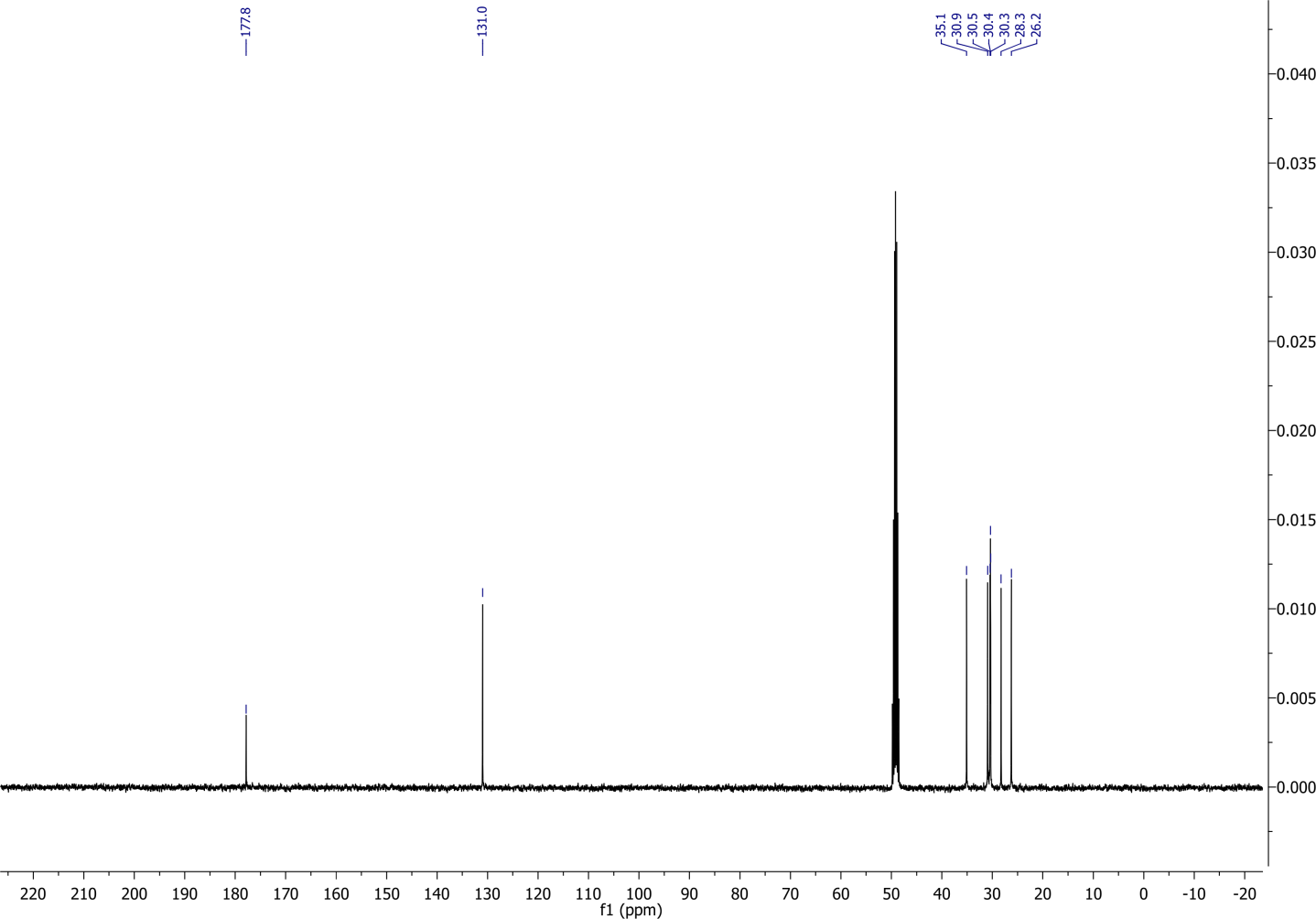


**Supplimentary Figure S10:** ^13^C-NMR of 1,18-*cis*-Octadec-9-enedioic acid (100 MHz, CD_3_OD): 177.8, 130.9, 35.1, 30.9, 30.5, 30.37, 30.3, 28.3, 26.2.

**Original figures**

Figure 3

| Figure after editing by cropping, splitting color channels and insertion of size bar using ImageJ 1.51d software (refer also to the methods) | Figures before editing | | |
| --- | --- | --- | --- |
| 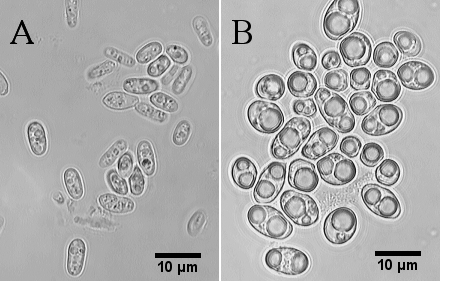 | A | 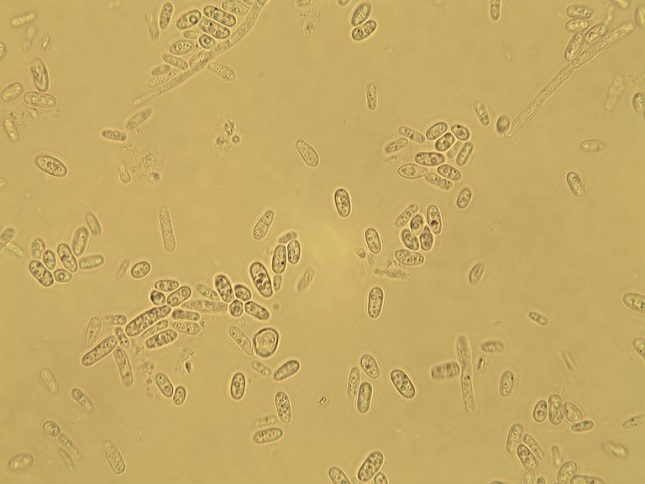 |  |
|  | B | 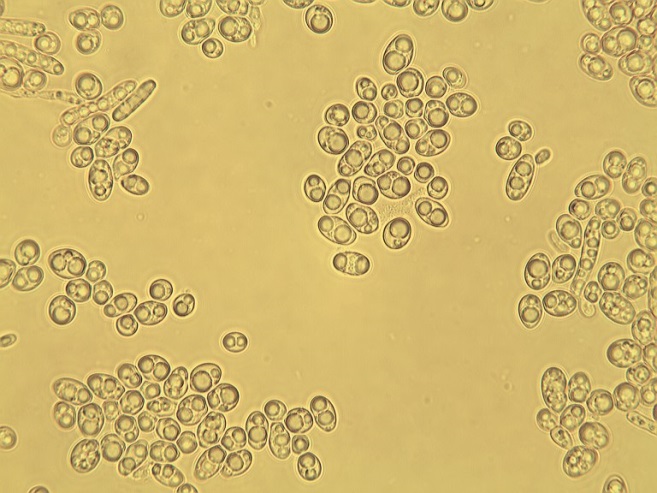 |  |

**Figure 4**

| Figure after editing by cropping, labelling the lines and splitting color channels using ImageJ 1.51d software (refer also to the methods) | Figure before editing |
| --- | --- |
| 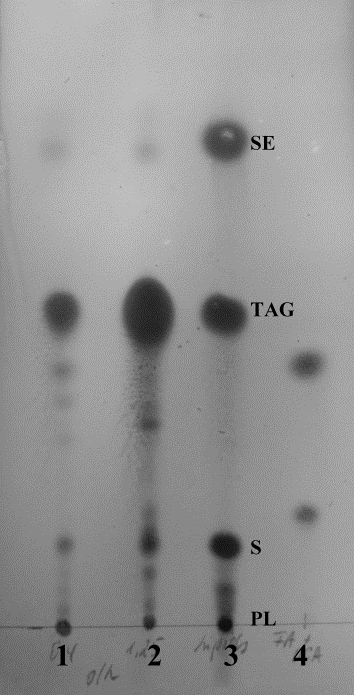 | 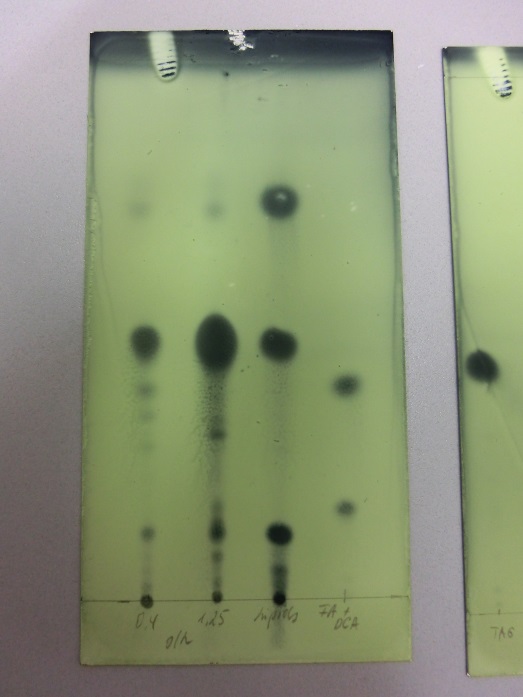 |

Supplementary Figure S6

| Figure after editing by cropping, splitting color channels and insertion of size bar using ImageJ 1.51d software (refer also to the methods) | Figure before editing |
| --- | --- |
| 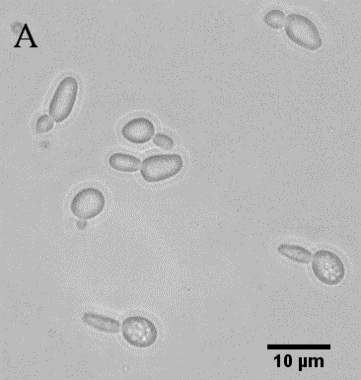 | 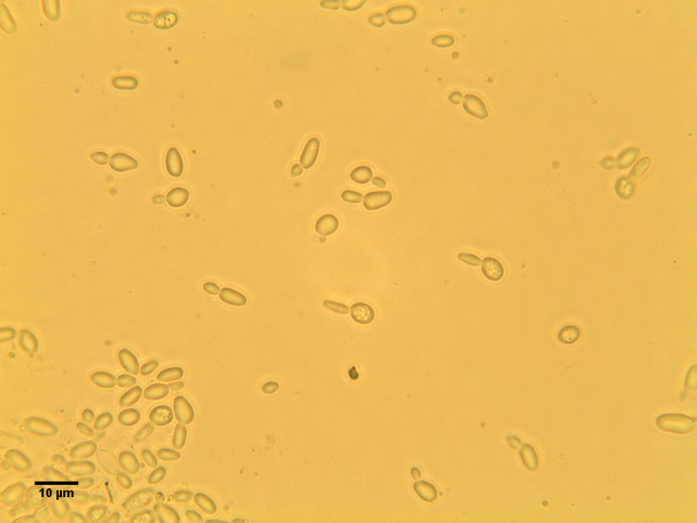 |
